# La coqueluche dans le monde. Vacciner l'enfant et l'adulte

**DOI:** 10.48327/mtsi.v3i4.2023.446

**Published:** 2023-11-22

**Authors:** Dominique GENDREL, Josette RAYMOND

**Affiliations:** Université Paris Cité, 12 de l’École-de-Médecine, 5006 Paris, France

**Keywords:** Coqueluche paucisymptomatique, Portage asymptomatique, Vaccin acellulaire, résurgence de la coqueluche, Allèle *ptxPl*, Paucisymptomatic pertussis, Asymptomatic carriage, Acellular vaccine, Pertussis resurgence, *ptxPl* allele

## Abstract

La coqueluche est une cause importante de morbidité et de mortalité infantile dans le monde et demeure un problème de santé publique malgré des niveaux souvent élevés de couverture vaccinale chez le nourrisson. La maladie, causée par la bactérie *Bordetella pertussis,* est présente dans tous les pays.

La vaccination est remarquablement efficace, avec une chute impressionnante des décès passant pour l'OMS de 4 millions/an en 1950 à 100 000/an en 2015, en grande majorité avant 2 ans dans les pays du Sud. Mais la morbidité reste importante, signe d'une circulation persistante de la bactérie, même avec des couvertures vaccinales élevées chez le jeune enfant. La principale raison est que les rappels sont insuffisants chez le grand enfant et l'adulte, que les pays utilisent le vaccin à germe entier ou le vaccin acellulaire. Les progrès majeurs sont la diffusion de la PCR, qui permet un diagnostic précis et rapide, et la vaccination de la mère au dernier trimestre de la grossesse pour que les anticorps transmis protègent le jeune nourrisson. La généralisation du vaccin acellulaire dans les pays occidentaux a été suivie d'une résurgence des cas de coqueluche progressant constamment. Qui plus est, on décrit maintenant en Chine et dans les pays voisins l'apparition de l'allèle *ptxPl,* souvent associé à une résistance aux macrolides. L'augmentation de cet allèle, rare auparavant, fait suite à la diffusion du vaccin acellulaire et concernerait plus de 30% des souches isolées chez l'enfant.

Il est donc indispensable de conserver les différentes souches pour des études génotypiques et d'antibiorésistance. Le plus important est de respecter le calendrier vaccinal chez l'adolescent et l'adulte qui sont les principaux contaminateurs.

## Abstract (en)

Pertussis (whooping cough) is an important cause of morbidity and mortality in infants world-wide, and continues to be a public health concern despite high vaccination coverage. The disease, caused by bacterium *Bordetella pertussis,* is present in all countries. Before vaccines became widely available in the 1950s, pertussis was one of the most common childhood diseases worldwide. According to WHO, estimation of deaths was 4 millions/year in 1950 and 100 000/year in 2015. But morbidity remains important with a high circulation of the bacterium determining atypical clinical forms after whole cell or acellular vaccines use. This is due mainly to the absence of booster doses in adolescents and adults. Major progress are generalisation of PCR and vaccination of mother during pregnancy. A resurgence of pertussis is observed after generalisation of acellular vaccines use. In China the progression of allele *ptxPl* was found in all areas following the use of acellular vaccine. This allele, rare before acellullar vaccine, is linked to a macrolide resistance, and reaches more than 30% of strains isolated in hospitalised children.

These evolutions must be evaluated in clinical forms and genotyping of all strains, in all areas.

## Introduction

La coqueluche, maladie strictement humaine à transmission respiratoire, a remarquablement bénéficié de la vaccination débutée dans la seconde moitié du siècle dernier. L'OMS estime qu'en 1950 (pour une population mondiale de 2,5 milliards d'individus) les décès par coqueluche chez les enfants de 0 à 5 ans étaient de 4 millions/an, contre 60 000 à 100 000 en 2020 pour 7,5 milliards d'individus [5, 7, 24]. **L'effet de la vaccination a été spectaculaire** sur le nombre considérable de décès et encore plus dans la forme la plus grave de la maladie chez le nourrisson, c'est-à-dire avec quintes et reprise inspiratoire bruyante (le chant du coq) longue et parfois fatale. Aux États-Unis, le taux d'attaque des coqueluches typiques passait de 150 à 200/10 000 dans les années 1940 à 0,5 à 1 dans les années 1970. En France, on estimait en 1950 le nombre des coqueluches cliniques à 7000 par an avec 200 décès, alors qu'en 1970, les chiffres étaient tombés respectivement à 1500 et 20. En 1986, année où la France a décidé d'interrompre la surveillance active de la coqueluche, on n'observait plus que quelques dizaines de cas de formes typiques avec moins de 5 décès par an [11, 28].

Mais ces résultats concernent les pays occidentaux où le taux de couverture vaccinale dépasse régulièrement 90% chez l'enfant de 2 ans. De plus, toutes les coqueluches, typiques comme paucisymptomatiques, y sont facilement diagnostiquées par la PCR (Polymerase Chain Reaction) disponible partout [5, 7, 11, 24]. Dans les pays à revenus faibles et intermédiaires (PRFI), la couverture vaccinale est très hétérogène. Elle peut atteindre des chiffres du même ordre dans les milieux privilégiés avec une forte présence médicale. Mais elle reste toujours insuffisante dans les villes, les banlieues et les régions éloignées, malgré de réels progrès soulignés à la fois par l'OMS et le GPI (Global Pertussis Initiative). Plusieurs enquêtes prospectives, certes rares et limitées, dans des régions isolées de différents pays où des efforts vaccinaux sont réels, ont montré une réduction nette des cas de coqueluche [5, 18, 24].

Le principal problème pour réussir à mesurer l'efficacité vaccinale et la circulation de germe dans ces pays, récemment encore appelés « tiers-monde », est l'absence de disponibilité de la PCR, seul moyen d'avoir un diagnostic rapide et précis de l'infection, la culture étant longue et difficile. Les formes paucisymptomatiques sont fréquentes dans l'entourage familial au sens large, surtout chez la mère mais aussi chez l'adolescent et l'adulte. Ces infections coquelucheuses quel que soit l’âge, même réduites à un portage quasi asymptomatique sont toujours contagieuses. Plusieurs études menées avec PCR au Maroc et en Iran montrent bien que, comme en Occident, ces formes mineures sont fréquentes chez la mère et dans l'entourage familial. Cette estimation de la circulation du germe, et donc de la définition des programmes vaccinaux après l’âge de 10 ans, n'est possible qu'avec la généralisation de la PCR [19, 29, 30].

La mortalité par coqueluche concerne avant tout, mais non exclusivement, le nourrisson de moins de 1 an. Il est donc indispensable de la distinguer des autres infections respiratoires sévères, en particulier celles dues à des germes à prévention vaccinale, pneumocoque et *Haemophilus* en particulier, pour déterminer un calendrier adapté.

Avant de considérer les aspects actuels de l'infection et les différentes propositions vaccinales, il importe de réfléchir sur la place des progrès réalisés en matière de diagnostic et de prévention, ainsi que sur les données épidémiologiques apparues récemment. Ces évolutions importantes de la clinique et de la lutte ne concernent pas seulement les pays industrialisés en Occident, mais nécessitent une surveillance avec des modalités adaptées aux pays à revenus moyens ou franchement faibles (PRFI).

## Données récentes

### Diagnostic par PCR et vaccination anténatale sont les progrès majeurs [5, 7, 11, 18, 24]

Ce sont de très loin en matière de diagnostic et de prévention les acquis récents les plus importants. Ils doivent se répandre partout et il faut mieux informer les soignants comme les familles sur les aspects connus et méconnus de la coqueluche.

**L'amplification génique par PCR** (Polymerase Chain Reaction) est la méthode diagnostique indispensable pour identifier le germe, la culture restant longue et difficile. L'accès à la PCR sous ses différentes formes, quels que soient les systèmes de santé et les revenus des pays, même dans des zones isolées, est important selon des modalités à définir. Cette utilisation de la PCR est considérée comme indispensable à la fois par l'OMS et le GPI (Global Pertussis Initiative), et tous les efforts doivent porter sur sa généralisation.

La mortalité par coqueluche est observée principalement avant 1 an et surtout dans les premiers mois de vie. La vaccination avant 3 mois restant largement inefficace en raison de la faible réponse immunitaire, la **vaccination anticoquelucheuse au dernier trimestre de la grossesse** s'impose [6, 8, 29]. Cette recommandation est considérée comme essentielle pour tous les pays, tant par l'OMS que par le GPI ou le CDC, quelle que soit la couverture vaccinale. Les premières estimations globales montrent que les résultats sont remarquables tant dans les pays industrialisés que les PRFI.

### Le vaccin acellulaire et ses conséquences possibles [5, 24, 29]

Il représente un progrès incontestable car il est mieux toléré que le vaccin à germes entiers, même si sa durée de protection est un peu moins longue. Il est diffusé uniquement et utilisé de façon exclusive dans les pays industrialisés et en Chine. Les autres pays, c'est-àdire la majorité de la planète, ont conservé le vaccin à germes entiers.

#### 1) Émergence de nouveaux génotypes et vaccins acellulaires

Les données exposées ici sont parfois discutées car elles ne concernent pour l'instant que plusieurs pays de l'Est asiatique. Mais en Chine les études sont importantes, cohérentes et fiables, appliquant des techniques reconnues par tous [3, 14, 15, 17]. Elles ont été effectuées dans des laboratoires contrôlés indépendants les uns des autres et répartis sur tout le territoire chinois. Leurs résultats concernent une population de plusieurs millions d'enfants atteints de coqueluche hospitalisés.

Ces études montrent toutes l’émergence d'un génotype *prn1/ptxP1/ptxA1/fim3-1/fim2-1* de *B. pertussis* présentant une résistance aux macrolides associée. Cette résistance est due à une mutation du gène codant pour l'ARN 23 S (A2047G). Cette émergence rapide de ce génotype et de cette association avec le gène *ptxP1* (allèle du promoteur de la toxine de *B. pertussis, ptxP* et à l'origine d'une résistance aux macrolides). est inquiétante. Surtout, cette émergence a été observée dans les années qui ont suivi la généralisation du vaccin acellulaire en Chine mais pas au temps du vaccin entier [3, 14]. Cette coïncidence, doit être absolument recherchée dans d'autres pays utilisant des vaccins acellulaires autres que le vaccin chinois

En Europe et en Amérique du Nord, les souches étudiées depuis plus de 30 ans portent pratiquement toutes l'allèle *ptxP3* et aucune résistance aux macrolides ou aux quinolones n'est signalée. Cependant très peu de travaux précisant la répartition *ptxP1/ptxP3* dans les pays occidentaux utilisant le vaccin acellulaire n'ont été publiés ces 5 dernières années.

L'apparition récente et importante en Chine de cet allèle *ptxP1,* le plus souvent associée à une résistance aux macrolides, doit être considérée comme l’évènement génotypique majeur, conséquence rapide de la généralisation du vaccin acellulaire. Cet allèle serait retrouvé actuellement dans 20 à 30% des souches étudiées chez les enfants chinois hospitalisés pour coqueluche, sur l'ensemble du territoire. En d'autres termes, on peut considérer que cette mutation pourrait être présente chez plusieurs millions d'enfants vaccinés par le vaccin acellulaire, éventuellement porteurs asymptomatiques ou avec des signes mineurs, et répartis dans toute la Chine centrale. Elle diffuse rapidement dans des pays limitrophes du Sud-Est asiatique en particulier au Vietnam, avec la même résistance aux macrolides associée [14]. Diverses équipes ont récemment montré que cette augmentation de circulation de souches portant *ptxP1* avec une résistance aux macrolides, est maintenant présente en Inde et au Japon dans les zones utilisant le vaccin acellulaire.

Des cas ont été mis en évidence au Mexique dans des régions proches des États-Unis utilisant le vaccin acellulaire. Mais c'est principalement en Iran que la présence de l'allèle *ptxPl* a été retrouvée dans des communautés où l'utilisation du vaccin acellulaire est habituelle [23, 27].

Cet allèle était très peu fréquent en Iran dans les souches conservées datant de l'utilisation exclusive du vaccin à germes entiers, mais quelques cas sont apparus récemment [27]. L'apparition de *ptxPl* au Moyen-Orient comporte un risque de diffusion dans des régions proches, voire en Europe où le vaccin acellulaire est très largement utilisé.

Il n'existe d'autre part aucune donnée concernant une augmentation particulière de *ptxPl* en lien avec un type particulier de vaccin acellulaire, celui-ci ayant une composition variable selon les fabricants. Toutes les données confirment que la croissance continue et régulière du nombre de souches portant *ptxPl* est une conséquence de la généralisation du vaccin acellulaire.

Ces émergences génomiques pourraient concerner une population infantile très importante en Chine notamment, de l'ordre de plusieurs millions d'enfants car le vaccin acellulaire s'y généralise rapidement. Elles imposent dans les pays utilisant le vaccin acellulaire une surveillance de la sensibilité aux antibiotiques et des évolutions génotypiques des *B. pertussis.* La conservation des souches est donc indispensable, en particulier dans les zones avec généralisation totale, voire partielle, du vaccin acellulaire, quel que soit le niveau de développement du pays. Le regroupement est nécessaire dans des laboratoires de référence.

#### 2) Résurgence de la coqueluche et vaccin acellulaire [1, 5, 24]

On constate depuis une vingtaine d'années, c'est-à-dire depuis la diffusion large du vaccin acellulaire, une résurgence de la coqueluche, mais son importance, et, pour certains sa réalité, font débat. Il s'agit d'une augmentation du nombre de coqueluches souvent atténuées, confirmées par la PCR.

**Figure 1:**
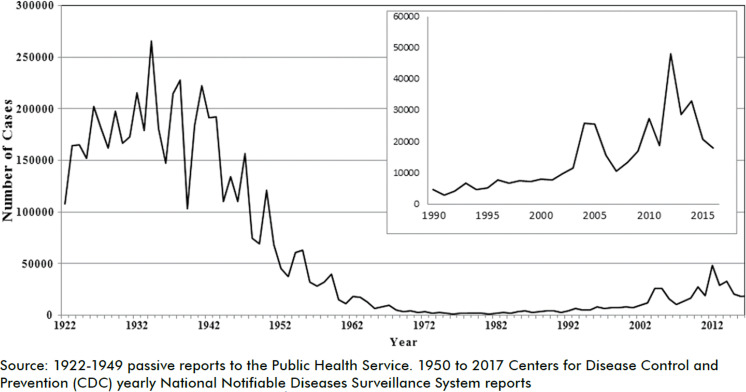
Cas de coqueluche recensés aux Etats-Unis Pertussis cases reported in the United States

La résurgence de la coqueluche après généralisation du vaccin acellulaire continue de progresser avec des formes peu sévères [5, 8, 29]. En Chine et aux États-Unis (Fig. 1 et 2), une surveillance attentive portant sur des populations très importantes, montre que l'augmentation progressive des cas se poursuit malgré une couverture vaccinale élevée de l'enfant avec vaccin acellulaire et démontre la progression de la circulation du germe dans la communauté.

**Figure 2:**
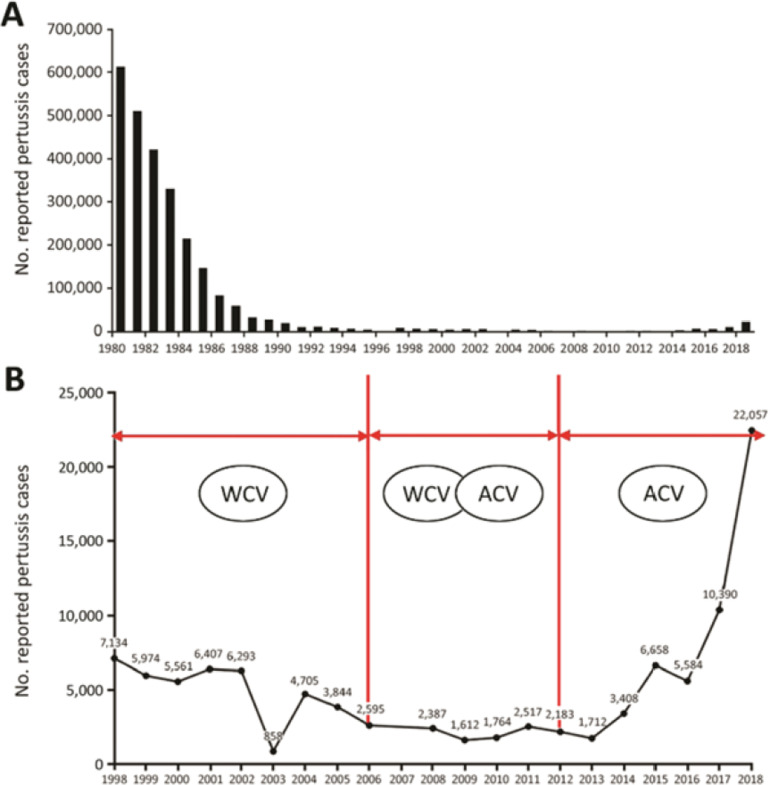
Cas de coqueluche recensés en Chine avec vaccin à germe entier (WCV) et acellulaire (ACV) [16] Pertussis cases reported in China with whole-cell (WCV) and acellular (ACV) vaccine [16]

Plusieurs explications ont été proposées. La première porte sur la durée de protection moins longue avec le vaccin acellulaire (6 à 8 ans) qu'avec le vaccin à germe entier (8 à 10 ans). Une autre plus convaincante de l'accroissement de la circulation du germe malgré une couverture vaccinale correcte de l'enfant jusqu’à 2 ans (DTP4 pour l'OMS) est que la protection serait insuffisante faute de rappels chez l'adulte et l'adolescent. Le rappel à l’âge de 5 ou 6 ans n'est pas toujours effectué, alors que, pour l'OMS et le GPI, c'est le rappel le plus important pour assurer une immunité prolongée chez le grand enfant et l'adolescent. Les rappels vaccinaux à 10 et 15 ans sont souvent négligés, voire non recommandés dans certains calendriers vaccinaux.

Les épidémies californiennes dans les écoles [31] ont montré que les nombreux collégiens atteints, d’âge compris entre 11 et 16 ans n'avaient pour la plupart pas eu de rappel à 5 ans et aucun à 10 ans. Partout dans le monde la vaccination anticoquelucheuse du grand enfant et de l'adolescent est trop souvent insuffisante, favorisant ainsi la circulation de germe [18, 19].

C'est dire que la morbidité réelle de la coqueluche, maladie strictement humaine, est totalement sous-estimée car on évalue très mal le nombre de coqueluches mineures ou atypiques et pas du tout le portage simple, sources majeures de la contagion. Sans la PCR qui permet d'identifier les nombreuses variations cliniques, on en resterait, faute de mieux, à la définition ancienne de la coqueluche pour l'OMS et le CDC: toux de plus de 21 jours pour l'une, de 14 jours pour l'autre.

## La Bactérie et le diagnostic de la coqueluche

### La bactérie

La coqueluche est une maladie uniquement humaine hautement contagieuse due à *Bordetella pertussis* ou bacille de Bordet-Gengou, bactérie à Gram négatif se transmettant par voie respiratoire. *Bordetella parapertussis,* qui n'est impliqué que dans 2 à 4% des coqueluches, n'exprime pas toutes les toxines de *Bordetella pertussis* et les patients infectés ont une clinique modérée. Mais des épidémies d'ampleur limitée, et surtout des cas isolés, à *Bordetella parapertussis* sont plus fréquentes depuis l'introduction du vaccin acellulaire. On peut quelquefois retrouver dans le rhinopharynx des enfants ou des adultes *Bordetella holmesii,* bactérie impliquée dans les déficits immunitaires. *Bordetella bronchiseptica* est une bactérie animale exceptionnellement isolée dans les prélèvements respiratoires humains.

*Bordetella pertussis* est sensible à beaucoup d'antibiotiques et principalement aux macrolides, employés habituellement chez le patient et son entourage, permettant une éradication du rhinopharynx en 3 jours. Mais cela pourrait évoluer avec l'apparition récente de souches résistantes aux macrolides associées à l’émergence de *ptxPl.*

### Toxines et adhésines

La pathogénie de la coqueluche est due aux toxines exprimées par *Bordetella pertussis.* La principale est la toxine pertussique (PT) à l'origine de la toux quinteuse et de l'hyperlymphocytose au cours de l'infection. Tous les vaccins contiennent la PT comme antigène. *Bordetella parapertussis,* germe proche qui ne produit pas de PT, ne donne qu'une coqueluche atténuée. Les adhésines sont des protéines de paroi du germe qui participent à la colonisation des cellules respiratoires. Elles sont immunogènes et constituent des éléments des vaccins acellulaires. Les principales sont l'hémagglutinine filamenteuse et les protéines fimbriales. On en rapproche l'adényl-cyclase et la pertactine. L'infection due à des souches de *Bordetella pertussis* non sécrétrices de pertactine a parfois une clinique modérée [15] mais cela n'a pas été confirmé. Cependant, des études protéomiques récentes [20] laissent supposer que certaines adhésines pourraient évoluer et participer à la virulence de la bactérie.

## Diagnostic biologique et performance des tests

### Diagnostic biologique

La performance des tests pour le diagnostic de la coqueluche dépend du stade de la maladie. Leur but est d'identifier à partir d'un prélèvement respiratoire *B. pertussis* dès les premières semaines de la maladie, soit par recherche de matériel génétique de la bactérie par Polymerase Chain Reaction (PCR), soit plus difficilement, par la culture de la bactérie.

#### 1) Amplification génique ou PCR

La PCR permet de détecter la présence de *Bordetella* jusqu’à plus de 3 semaines après le début de la toux à partir d'un prélèvement nasopharyngé (écouvillonnage ou aspiration). Deux techniques peuvent être utilisées:

- la PCR en temps réel (RT-PCR)
  La principale cible utilisée pour la RTPCR du diagnostic de *B. pertussis* est la séquence d'insertion *IS481* qui présente une grande sensibilite du fait d'un grand nombre de copies dans le génome (> 240). Afin de spécifier la nature de la *Bordetella* en cause, des PCR complémentaires utilisant d'autres cibles peuvent être réalisées pour *B. pertussis, B. parapertussis et B. holmesii.*
- les PCR syndromiques
  Il s'agit de panels syndromiques basés sur la détection par PCR de plusieurs pathogènes respiratoires, bactériens et viraux. Ils sont disponibles partout et largement commercialisés. Parmi eux, le plus utilisé est le panel respiratoire FilmArray Mérieux qui est un test de diagnostic multiplex *in vitro* permettant la détection simultanée de 22 pathogènes respiratoires (18 virus et 4 bactéries dont *B. pertussis)* à partir d’échantillons rhinopharyngés. En ce qui concerne *B. pertussis,* ce test ne détecte que les charges bactériennes importantes.
  D'autres panels avec PCR multiplex sont commercialisés, de provenances diverses, mais peu testés par les laboratoires de référence. Malgré leurs limites, ces kits de PCR syndromique sont remarquablement utiles, d'un prix accessible et surtout permettent la recherche d'un nombre important de différents pathogènes respiratoires.

#### 2) La culture

La culture pourrait être le test de référence pour le diagnostic de la coqueluche si elle n’était aussi longue et difficile. Sa sensibilité est de 50 à 60% lors de la première semaine de toux, puis elle diminue rapidement. Sa spécificité est excellente. Elle est « fastidieuse » mais indispensable pour étudier les souches, leur résistance aux antibiotiques et leur évolution génomique. Les souches isolées doivent être envoyées aux centres de référence pour regrouper les études génomiques.

#### 3) La sérologie

La sérologie n'a plus sa place dans la stratégie diagnostique de la coqueluche. Elle présente une très mauvaise spécificité, et une hétérogénéité de performance en fonction des kits utilisés. La seule technique recommandée est la technique immuno-enzymatique ELISA (Enzyme-Linked Immunoabsorbent Assay) utilisant de la toxine de pertussis hautement purifiée.

Une coqueluche aiguë ne peut être diagnostiquée qu'avec l’élévation du taux d'anticorps à deux prélèvements successifs séparés de deux semaines.

L'OMS limite son utilisation, faute de mieux, aux enquêtes de prévalence sur une large population [24].

## Infection à *Bordetella Pertussis*

La coqueluche « classique » est toujours d'actualité et continue d’être enseignée, même si la symptomatologie complète est rare. Les coqueluches mal tolérées ou sévères sont hospitalisées, et sont en principe comptabilisées dans les bilans de surveillance de la maladie, contrairement aux formes ambulatoires mineures.

Les portages asymptomatiques sont connus avant tout par les bilans par PCR autour du cas index. Mais nous manquons d'enquêtes systématiques dans la communauté pendant ou en dehors des périodes épidémiques, tant dans les pays occidentaux que les PRFI [10, 12, 13, 18, 23]. Le diagnostic d'un cas de coqueluche impose de traiter immédiatement par antibiotiques, généralement des macrolides, la famille, comme tout l'entourage au sens large sans demander de test. Trois jours d'azithromycine est le traitement habituel [24, 28].

## La maladie coquelucheuse [1, 4, 5, 18, 19, 28]

La maladie provoquée par *Bordetella pertussis* évoluerait par cycles tous les 3 à 5 ans, peu nets si la couverture dépasse 50% [5, 24]. Elle présente des caractéristiques qui la différencient des autres infections respiratoires sévères observées chez l'homme. Le signe principal est une toux paroxystique non productive survenant par quintes avec reprise inspiratoire bruyante, épisodes souvent nocturnes séparés par des périodes de normalité respiratoire.

L'infection est non fébrile. La fièvre ne fait pas partie du tableau clinique. Sa présence au cours de la coqueluche doit faire rechercher une infection associée à d'autres pathogènes, bactéries ou virus.

Classiquement, on décrit chez l'enfant non vacciné, après une incubation silencieuse de 7 à 10 jours, une phase catarrhale d'une semaine avec rhinorrhée et toux. La phase suivante paroxysmale de 2 à 4 semaines est marquée par une toux émétisante, souvent nocturne, avec quintes et reprise inspiratoire cyanogène bruyante (le chant du coq) souvent mal supportée, et un risque d'apnées sévères. La phase de convalescence avec décroissance progressive de la toux peut durer plusieurs semaines. Chez les enfants vaccinés avec une seule dose, le tableau est moins sévère.

Pour éviter la contagion, il faut systématiquement traiter tout l'entourage par 3 jours d'azithromycine sans demander d'examens de confirmation et mettre à jour le calendrier vaccinal chez les proches.

## La coqueluche du nourrisson et de l'enfant et la contamination familiale [2, 6, 23, 29]

Les formes sévères de coqueluche et les décès s'observent avant l’âge de 1 an chez des nourrissons non vaccinés. Il s'agit toujours d'une contamination familiale.

La prescription systématique de macrolides au patient et à la famille réduit considérablement la contagion, et permet de lever l'interdiction de visites après 3 jours de traitement.

La toux paroxystique et émétisante, suivie d'une reprise inspiratoire cyanogène bruyante est dangereuse car une apnée avec arrêt cardio-respiratoire brutal est toujours menaçante. Les apnées font toute la gravité de la coqueluche du nourrisson. Elles peuvent sembler isolées tant l’épisode de toux qu'elles suivent est parfois bref.

La transmission de la coqueluche chez le nourrisson est exclusivement familiale, et dans plus de la moitié des cas il s'agit de la mère, souvent porteuse asymptomatique. C'est un argument majeur pour la vaccination pendant la grossesse car le nouveau-né est alors protégé par les anticorps maternels transmis à travers le placenta, et surtout la mère, potentiellement transmettrice, est protégée de l'infection par la vaccination [2, 5, 12, 13, 22].

**Figure 3:**
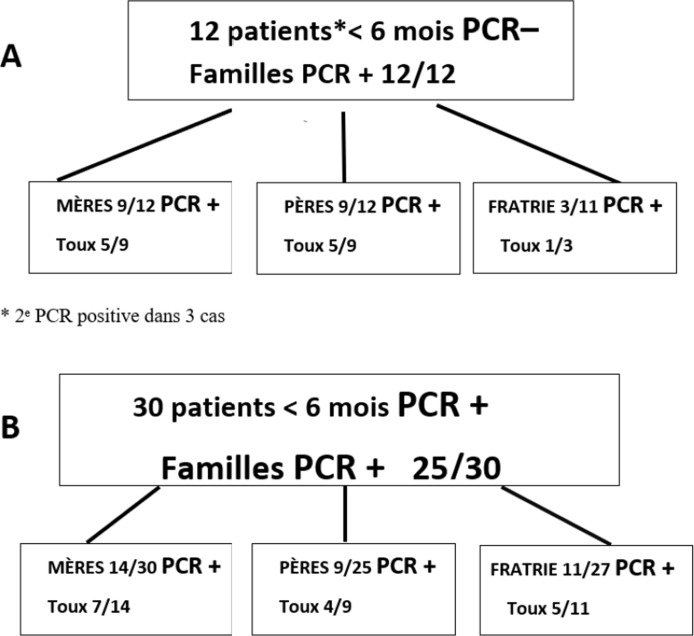
Enquête familiale autour d'un cas chez 42 nourrissons < 6 mois hospitalisés pour coqueluche (Hôpital Saint-Vincent-de-Paul, Paris, 2005-2006) [26] A: PCR initiale négative chez le cas index mais positive chez au moins 1 membre de la famille B: Cas index avec PCR positive PCR results in families of 42 infants (< 6 months) hospitalized for pertussis in Paris Saint-Vincent-de-Paul hospital, 2005-2006. [26] A: First PCR negative in the index-case but positive in at least 1 family member B: PCR positive in index-case

La Figure 3 montre, dans un hôpital parisien, l'exploration autour d'un cas index de l'entourage familial complet et détaillé. Elle est exposée ici car elle détaille les formes cliniques et les portages de l'entourage, sources possibles de contagion. Les résultats marocains, iraniens et sud-africains sont identiques avec des formes mineures dans l'entourage [2, 5, 12, 13, 22]. Mais ces publications donnent trop peu de détails sur les membres de la famille proche et les personnes vivant au foyer qui ne sont pas tous explorés. Toutes les séries publiées, quel que soit le pays, montrent la même chose: c'est le plus souvent la mère qui contamine le nouveau-né, dans la moitié des cas par un portage simple sans toux. C'est pour cela que la vaccination en fin de grossesse est indispensable.

Il est fréquent de retrouver, avant l’âge de 1 an, une hyperlymphocytose souvent > 20 000. Elle n'est pas constante, mais c'est un excellent signe qui doit conduire à une PCR de coqueluche chez tout nourrisson tousseur et son entourage.

Des malaises graves du jeune nourrisson ou des apnées brutales, apparemment isolés, voire des morts subites peuvent être dus à la coqueluche avec absence de quintes typiques dans les jours précédents. Il faut toujours faire un prélèvement rhinopharyngé à ces enfants et à leur entourage pour rechercher la bactérie par PCR.

La **surinfection bactérienne** est fréquente, marquée par la fièvre qui doit alerter car la coqueluche n'est, en principe, pas fébrile. Les opacités parenchymateuses sur le cliché de thorax sont un bon signe, car une coqueluche non surinfectée ne montre que des signes radiologiques de type bronchique. Le plus souvent, il s'agit de pneumocoques.

L'association au Virus Respiratoire Syncytial (VRS) n'est pas rare chez le nourrisson de moins de 3 mois hospitalisé pour bronchiolite aiguë, mais elle peut être importante et compliquer l’évolution de la bronchiolite [2, 8, 26]. La coqueluche aggrave la situation et une assistance respiratoire en soins intensifs est souvent nécessaire. La coqueluche n'est que rarement évoquée car les signes de bronchiolite prédominent avec dyspnée expiratoire, toux et encombrement bronchique productif. Il est important de rechercher la coqueluche dans toutes les formes sévères de bronchiolite à VRS. De plus, il est indispensable de traiter l'entourage familial.

### La coqueluche de l'adolescent et de l'adulte

La clinique est très hétérogène et souvent peu spectaculaire car les patients ont une immunité résiduelle faible en ayant reçu une ou plusieurs injections vaccinales dans l'enfance. Le contact avec des patients ayant une coqueluche clinique peut augmenter l'immunité, mais c'est une situation assez rare si la population infantile est correctement, voire partiellement vaccinée. Dans tous les cas l'infection, typique ou non, témoigne d'une absence des rappels conseillés pendant l'enfance et l'adolescence [1, 10, 18, 24, 30, 31].

Les formes sévères conduisant à une hospitalisation sont rares et souvent liées à une pathologie sous-jacente, en particulier l'asthme. Des épidémies limitées sont possibles dans les communautés d'adolescents, camps de vacances, internats ou autres.

Les épidémies de 2010 et 2014 en Californie [29, 31] sont riches d'enseignement. Les données concernent plus de 900 patients avec PCR positive et un âge moyen de 10,5 ans. La couverture vaccinale par vaccin acellulaire avant 2 ans était de 90%, mais l'injection de rappel à 5 ans était inférieure à 60%. Il n'y avait pas de formes sévères ni d'hospitalisations liées à la coqueluche, mais 60% des patients positifs avaient une toux importante avec quintes et parfois vomissements, les autres avaient une toux banale. De plus, 8 à 10% de ces adolescents avec PCR positive n'avaient aucune toux ni d'autre manifestation clinique et étaient donc porteurs sains du germe mais contagieux.

Ces résultats montrent que l'immunité par vaccin acellulaire peut être brève et que les rappels chez le grand enfant et l'adolescent sont indispensables. D'une façon générale, la couverture vaccinale demande à être très importante à tous les âges pour montrer son efficacité avec des protocoles adaptés aux conditions locales.

Le portage asymptomatique est possible partout. Une étude chinoise bien faite rapporte qu'en prélevant des élèves dans plusieurs écoles en dehors d’épidémies, on a trouvé par PCR, 5 à 8% de porteurs asymptomatiques [17]. De telles études sont trop rares mais indispensables dans les pays développés comme les PRFI.

L'infection par *Bordetella pertussis* de l'adulte est sous-estimée, car les recommandations vaccinales chez l'adulte sont généralement mal suivies, voire inexistantes. D'autre part, toutes les données sur l'entourage autour d'un cas index montrent bien que la fratrie, le père et les adultes présents au foyer, en particulier la mère, sont des transmetteurs fréquents avec des formes mineures ou un portage simple, sans toux (Fig. 3).

Plusieurs enquêtes réalisées en ambulatoire chez des adultes dans les années 2000 ont montré, en France, que 15 à 20% environ des patients de plus de 60 ans toussant plus de 7 jours sont infectés par *Bordetella pertussis.* Des chiffres identiques sont rapportés au Danemark et dans d'autres pays européens. Ces adultes sont donc contagieux [2, 12, 19, 30]. Tous les cas groupés de toux, même modérée, dans une communauté d'adultes, travail ou loisirs, doivent faire rechercher une coqueluche. Quelques données concernent les maisons de séjour pour personnes âgées: les patients toussent généralement peu mais de façon continue. Le personnel est souvent porteur sain ou avec une forme mineure. Les soignants, infirmières comme médecins, peuvent être à l'origine d'une transmission à leur famille, voire à leurs patients: les recommandations concernant l'adulte (vaccin à 25, 45 et 65 ans) doivent être strictement respectées [5, 8, 28].

Il persiste donc beaucoup d'inconnues sur l'infection à Bordetella de l'adulte. Elle est sous-estimée avant tout parce qu'elle n'est pas recherchée. En particulier on connaît trop peu son rôle dans l'aggravation d'une pathologie respiratoire chronique.

### La coqueluche dans les pays à revenus faibles et intermédiaires (PRFI)

C'est dans ces pays que la coqueluche est la plus importante, la plus dangereuse et la plus mal connue. En 2015, l'OMS donnait une fourchette très large pour les décès dans ces pays, de 100 000 à 300 000 par an. La maladie est insuffisamment étudiée et mal identifiée. La couverture vaccinale varie considérablement, entre 50 et 70% avant 2 ans en Afrique ou en Asie, mais 90% pour une classe aisée bien médicalisée.

Les études marocaines [13] et iraniennes [23, 27, 32], réalisées à la fois dans les villes et les campagnes, montrent bien cette hétérogénéité qui dépend avant tout du revenu familial, avec les formes les plus graves dans les zones les moins couvertes médicalement. Les études sud-africaines soulignent le caractère cyclique des épidémies, même avec une couverture vaccinale de l'ordre de 60%, et les atteintes les plus sévères chez les jeunes nourrissons [21, 22]. Le principal message est que tous les signalements même incomplets de coqueluche, comme des études les plus systématiques, sont indispensables. Son importance comme cause de mortalité avant 6 mois est majeure. C'està-dire que la vaccination de fin de grossesse est indispensable. Cependant, les anticorps transplacentaires ne vont persister que 3 mois. Dans de nombreuses régions africaines, il existe des placentopathies palustres limitant le transfert transplacentaire des anticorps [8, 9], la circulation de la bactérie est importante, les nourrissons de 3 à 18 mois sont à risque. Et nous avons bien vu que cette circulation dépendait largement des rappels vaccinaux de l'enfant à 5 ans en particulier et des rappels chez l'adolescent.

Dans les études réalisées dans différents pays non occidentaux, les formes mineures ou atypiques sont toujours présentes dans l'entourage familial et chez l'enfant infecté. Dans la communauté, le germe circule également: une étude africaine portant sur une cohorte d'enfants prélevés plusieurs fois montre que le portage asymptomatique n'est pas rare (10) Cela signifie que le germe circule de façon importante mais très inhomogène. Les quelques études sérologiques à large échelle montrent que les anticorps sont acquis rapidement par la vaccination et renforcés par le contact avec des patients infectés. L'interprétation de cette immunité dite naturelle doit être soigneusement appréciée en termes de protection contre l'infection. Quelques données collectées dans des régions isolées vont dans ce sens. Au Cameroun, plusieurs villages isolés et jamais vaccinés ont pu être étudiés avec la sérologie [18]. Plus de 80% des enfants entre 5 et 10 ans avaient des anticorps élevés, mais il persistait des cas de coqueluches graves chez les nourrissons. C'est-à-dire que la bactérie circulait toujours, malgré l'immunité acquise chez une partie de la population par contact avec les malades.

De toute façon, l'interprétation des résultats observés dans les pays à faibles revenus et dans les pays occidentaux va dans le même sens. La circulation de la bactérie et les contages persistent si les rappels chez l'adolescent voire l'adulte sont insuffisants, quel que soit le type de vaccin. Cela a été bien montré dans les pays occidentaux avec le vaccin acellulaire avec insuffisance des rappels, mais cela est tout aussi vrai pour le vaccin à germes entiers. L'exemple de la Pologne [19] le montre bien. Ce pays, bien sûr occidental et non tropical, est le dernier en Europe à utiliser le vaccin à germes entiers, sans effets secondaires notables, et la couverture vaccinale à 2 ans est de l'ordre de 90%. Mais les rappels sont conseillés à 8 et 14 ans seulement. La vaccination de la femme enceinte y est peu développée et le rappel à 14 ans parfois négligé. Depuis plusieurs années on constate en Pologne que les coqueluches du nourrisson persistent, alors que les coqueluches de l'adolescent sont devenues majoritaires et celles de l'adulte en augmentation. Il est certain que l'absence de vaccination systématique de la population infantile à 5 ans joue un rôle important [24]. C'est un argument supplémentaire pour adhérer à l'attitude de l'OMS qui considère que le rappel contre la coqueluche à 5 ans est fondamental pour limiter la circulation du germe.

Évaluer l'immunité générale, la circulation bactérienne et l'efficacité de la politique vaccinale est difficile mais indispensable. Il faut donc des moyens diagnostiques fiables pour les pays à faibles revenus.

Le plus important est d'implanter la PCR. Les prix sont trop élevés, mais par le biais des associations internationales ou humanitaires des commandes groupées sont possibles. Les PCR multiplex ou syndromiques, certes moins sensibles que la RT-PCR, trouvent là toute leur place. Le même appareillage, avec des réactifs différents, permettant également d'identifier plusieurs pathogènes respiratoires, constitue certainement le meilleur choix, d'autant que les différentes offres commerciales peuvent être mises en concurrence.

On ne doit pas séparer totalement les résultats des études centrées sur la coqueluche et sa prévention dans des endroits très différents au Nord comme au Sud car, malgré des différences importantes, elles vont dans le même sens [5, 24]: la circulation persistante de la bactérie en l'absence de rappels. La surveillance précise de la maladie comme les collections de cas, même imprécises, les signalements partiels obtenus dans les pays occidentaux comme dans les pays à revenu plus faible sont informatifs.

Si la vaccination de la femme enceinte est le progrès majeur en termes de protection, il reste à préciser l'importance des formes mineures à tous les âges. Il reste surtout à définir, de façon adaptée à l'environnement mais avec précision, le calendrier vaccinal du grand enfant et surtout de l'appliquer. De même, les rappels chez l'adolescent comme chez l'adulte sont indispensables mais restent insuffisamment recommandés dans les pays occidentaux comme dans les pays du Sud. La prévention vaccinale répétée est le seul moyen d’éviter une circulation persistante de la bactérie et le risque de contamination.

## Les vaccins et le calendrier vaccinal: de l'enfant à l'adulte

### Les vaccins à germes entiers et acellulaires

Il n'existe pas de valence anticoquelucheuse isolée dans les vaccins disponibles pour le public, tant acellulaires qu’à germe entier: toutes les spécialités commercialisées sont associées à d'autres valences vaccinales, au moins anatoxines diphtérique et tétanique, et comportant ou non les différentes valences poliomyélite. Les vaccins se présentent sous forme liquide de 0,5 ml et ne doivent pas être congelés.

Les vaccins à germes entiers sont préparés à partir de cultures de souches sélectionnées de *B. pertussis* qui sont ensuite tuées, habituellement par chauffage ou par traitement au formol. Chaque lot de vaccins subit des tests approfondis pour évaluer l'activité, la toxicité, la stérilité et la concentration en bactéries des vaccins. Les méthodes de production varient selon les fabricants, d'où une relative hétérogénéité, mais tous sont conformes aux recommandations de l'OMS.

Ils ne sont pas commercialisés dans les pays occidentaux, mais sont utilisés dans le monde par 60% des pays (le vaccin DTP classique). Il n'existe pas de vaccins à germes entiers à doses réduites pour le jeune enfant, comme nous en disposons pour les vaccins acellulaires.

Ils sont mieux tolérés que les vaccins à germes entiers produits dans les années 50 à 80. Les convulsions fébriles suivant l'injection sont très rares, un peu plus fréquentes qu'avec le vaccin acellulaire. La crainte des complications neurologiques précoces et tardives a été dans les années 70-80 la principale cause de la méfiance envers ces vaccins contre la coqueluche. Les refus n’étaient pas rares et l’évolution vers un vaccin mieux supporté s'imposait. Mais plusieurs études précises réalisées au Royaume-Uni avec un suivi prolongé, ont montré qu'il n'y avait pas de risque d'encéphalopathie précoce ou tardive dans les suites du vaccin à germe entier, ce que souligne l'OMS.

Les principaux effets secondaires du vaccin à germes entiers restent les réactions locales souvent importantes au point d'injection. Une rougeur douloureuse de 5 à 10 mm pouvant persister plusieurs jours est observée dans 60 à 80% des cas, avec une tolérance variable. Chez 10 à 15% des patients il s'agit d'un œdème régional important, allant parfois jusqu’à limiter la mobilité du membre concerné pendant quelques jours.

L'efficacité globale des vaccins à germes entiers est légèrement supérieure à celle des vaccins acellulaires en raison d'une durée de protection un peu plus longue. Dans un certain nombre de cas, les deux types de vaccin sont utilisés alternativement chez le même individu. Beaucoup considèrent que c'est acceptable quand on ne peut faire autrement, mais il n'existe pas d’études précises sur le passage d'un type de vaccin à l'autre chez le même enfant.

Les vaccins acellulaires contiennent un ou plusieurs antigènes purifiés: toxine pertussique, hémagglutinine filamenteuse, pertactine et fimbriae de types 2 et 3. Ils diffèrent non seulement par le nombre des composants antigéniques, généralement 3 [TP, HAF et PRN] ou 5 [TP, HAF, PRN et FIM] et par leur concentration, le clone bactérien utilisé pour la production, et les méthodes de purification et de détoxification. Ils peuvent donc varier dans leur composition selon le fabricant, sans que des études précises aient montré des différences d'efficacité.

Il existe des vaccins acellulaires à concentrations moins élevées, de type « dTcaPolio ou dTca » car ils comportent des doses réduites d'anatoxine diphtérique (d et non D) et d'antigènes coquelucheux acellulaires (ca et non Ca). La primo-vaccination et son rappel, avant 2 ans, est effectuée en principe avec dTca avec ou sans la valence polio. Mais la vaccination à 6 ans doit être effectuée avec un vaccin à doses entières soit DTCa.

La femme enceinte est vaccinée avec dTca ou dTcaP au deuxième ou troisième trimestre de la grossesse et lors des grossesses ultérieures (selon la HAS, l'OMS et le CDC).

Quant aux vaccins par voie nasale, les premiers résultats ont été décevants. Cependant, des travaux plus récents ont montré une action sur le portage par les cellules respiratoires.

### Le calendrier vaccinal

Le calendrier vaccinal habituellement recommandé (OMS, CDC), tant avec le vaccin entier qu'avec le vaccin acellulaire, est une primovaccination à 3, 4 et 6 mois suivie d'un rappel à 18 mois (DTP4) et de deux autres, à 5-6 ans et à 11 ans, avec vaccination au deuxième ou troisième trimestre de la grossesse. C'est le schéma choisi par la plupart des pays et il confère une forte immunité dès 1 an [5, 6, 7, 8, 24, 28].

Plusieurs pays, dont la France, ont choisi une primo-vaccination à 3 doses. Les injections sont programmées à 2, 4 et 11 mois, ce qui entraîne à l’évidence un moindre coût. Quelques coqueluches de forme mineure avant 5 ans sont possibles avec ce calendrier, comme le démontrent la surveillance clinique et la modélisation [25, 28]. Mais ce schéma 2-4-11 ne peut être appliqué que dans les pays bénéficiant d'une forte couverture vaccinale, supérieure à 97% avant 2 ans, du fait d’échecs possibles [25].

De toute façon, quels que soient le calendrier choisi et le type de vaccin, **le rappel à 5 ou 6 ans est indispensable.** On a montré, par la surveillance clinique et la modélisation, que ce rappel réduit la contagion dans la communauté. L'OMS souligne qu'il représente la meilleure stratégie de toutes les politiques vaccinales anticoquelucheuses pour protéger le grand enfant jusqu’à 12 ans [24].

Le calendrier des rappels varie d'un pays à l'autre. La plupart recommandent de combiner les rappels de la coqueluche avec les rappels des autres maladies (Diphtérie, Tétanos, Poliomyélite). Mais les calendriers nationaux concernant les rappels de l'adolescent et encore plus de l'adulte sont loin d’être semblables partout. Dans certains pays le premier rappel après 18 mois est même proposé à 8 ans ou à 10 ans, alors que le rappel à 5 ans est indispensable.

La plupart des pays recommandent un rappel à 11 ans, ou entre 10 et 15 ans. D'autres recommandent une seule dose de rappel entre 15 et 18 ans.

Les recommandations françaises et de plusieurs pays européens sont d'un rappel à 6 ans avec un vaccin à doses entières d'anatoxine diphtérique et de coquelucheux acellulaire (DTCa) suivi à 11-13 ans d'un rappel avec dTca ainsi qu’à 25 ans, en ajoutant une valence polio, selon les recommandations nationales. Si le rappel n'a pas été fait à 25 ans, il peut être rattrapé jusqu’à 39 ans révolus avec dTcaP. Une dose à 45 et à 65 ans est habituellement proposée.

La plupart des sociétés d'infectiologie, nationales et internationales, recommandent de suivre le schéma américain pour l'adulte, soit une dose tous les 10 ans à partir de 20-25 ans. **La vaccination dans les 6 derniers mois de la grossesse** est faite avec dTca ou dTcaP. Elle concerne la grossesse en cours et les grossesses ultérieures. Aucune recommandation particulière n'existe concernant le nombre de grossesses successives à vacciner, beaucoup s'arrêtent à 3 grossesses.

**Tous les professionnels de santé,** les personnes s'occupant de la petite enfance et des patients âgés en EPHAD, et les étudiants du secteur médical, infirmier ou autre doivent recevoir une dose de vaccin coquelucheux acellulaire et les rappels à 25, 45 et 65 ans (dTcaP).

Beaucoup de praticiens proposent une vaccination au père et à la fratrie si la dernière injection date de plus de 5 ans. Cette attitude date d'avant la vaccination systématique de la femme enceinte, mais reste utile dans de nombreux cas. La recommandation de la vaccination prénatale de la mère est récente en France, comme dans plusieurs autres pays européens [28]. En attendant qu'elle soit généralisée, le risque de contage familial doit être pris en compte.

### Le refus vaccinal

La principale limitation à l'emploi du vaccin anticoquelucheux est l'opposition aux vaccins. Le refus par les parents de la vaccination de leurs enfants cède la plupart du temps pour des raisons pratiques, c'est-à-dire les exigences pour fréquenter les collectivités. Les rappels chez l'adolescent et l'adulte, dont nous avons vu l'importance, peuvent être refusés avec très peu de moyens de pression. Quant à la vaccination de la femme enceinte, beaucoup de patientes la craignent. C'est dire toute l'importance, pour le vaccin anticoquelucheux comme pour tous les autres vaccins, d'une large information démontrant son importance pour la protection individuelle et de la communauté. Il faut également souligner à chaque occasion l'absence d'effets secondaires importants.

**Le caractère obligatoire des vaccinations** pourrait passer pour une atteinte aux libertés individuelles, mais il représente une contrainte très utile pour l'ensemble de la population. Cette obligation existe dans plusieurs pays. L'exemple de la France montre son utilité [28]: onze vaccinations sont obligatoires avant l’âge de 2 ans (Coqueluche, Diphtérie, Tétanos, Poliomyélite, *Haemophilus influenza* B, Hépatite B, Pneumocoque, Méningocoque C, Rougeole, Oreillons, Rubéole). Ainsi le taux de couverture pour la coqueluche est estimé à plus de 97% à 2 ans. L'exigence des rappels à 6 et 11 ans pour entrer à l’école primaire et secondaire et dans les collectivités revient pratiquement à une obligation. Cela permet un taux de couverture vaccinale qui approche 95% à 10 ans.

## Conclusion

La coqueluche est une maladie qui a changé avec l'effondrement des décès du nourrisson grâce à la vaccination. Mais la morbidité, avec les formes mineures et les portages asymptomatiques, est trop mal connue. En particulier, nous manquons d'enquêtes précises sur l'aggravation des formes aiguës ou chroniques de maladies respiratoires par la coqueluche tant chez l'enfant que chez l'adulte. La circulation du germe reste importante, même si elle est peu spectaculaire dans les pays à couverture vaccinale importante.

Les mutations génotypiques, avec la croissance de *ptxPl,* et la résurgence de la coqueluche apparues avec la généralisation du vaccin acellulaire sont des évènements majeurs, ainsi que le lien apparu récemment entre *ptxPl* et la résistance aux macrolides. La surveillance des souches est indispensable, notamment le rapport *ptxP1/ptxP3* dans les pays vaccinant tant avec le vaccin entier qu'avec le vaccin acellulaire. La virulence du germe, la progression de génotypes inhabituels et la résistance aux antibiotiques, conséquence du passage au vaccin acellulaire, pourraient devenir importantes.

Il est impératif de continuer à rechercher, à cultiver et à conserver les souches de *B. pertussis* partout dans le monde, quel que soit le vaccin utilisé, afin de surveiller les modifications du génome. Cela permettra probablement d'analyser les transformations cliniques et biologiques qui sont la conséquence des politiques vaccinales actuelles, avant d'envisager la diffusion de nouveaux vaccins.

## Contributions des auteurs

Les auteurs ont contribué à ce travail de manière équivalente.

## Liens d'intérêts

Les auteurs ne déclarent aucun lien d'intérêts.
